# Multiplexed Electrochemical Detection of Yersinia Pestis and Staphylococcal Enterotoxin B using an Antibody Microarray

**DOI:** 10.3390/s100403351

**Published:** 2010-04-06

**Authors:** Jason Wojciechowski, David Danley, John Cooper, Nina Yazvenko, Chris Rowe Taitt

**Affiliations:** 1 Center for Bio, Molecular Science and Engineering, Bld. 30, US Naval Research Laboratory, 4555 Overlook Ave. SW, Washington DC 20376, USA; E-Mail: jason.wojciechowski.ctr@nrl.navy.mil; 2 CombiMatrix Corporation, 6500 Harbour Heights Pkwy., Suite #303, Mukilteo, WA 98275, USA; E-Mails: ddanley@combimatrix.com (D.D.); jcooper@combimatrix.com (J.C.); nina@combimatrix.com (N.Y.)

**Keywords:** CombiMatrix, microarray, biosensor, electrochemical detection, super avidin-biotin system (SABS), immunoassay, pathogen detection

## Abstract

The CombiMatrix antibody microarray is a versatile, sensitive detection platform based on the generation and transduction of electrochemical signals following antigen binding to surface antibodies. The sensor chip described herein is comprised of microelectrodes coupled to an adjacent bio-friendly matrix coated with antibodies to the biological pathogens *Yersinia pestis* and *Bacillus anthracis*, and the bacterial toxin staphylococcal enterotoxin B (SEB). Using this system, we were able to detect SEB and inactivated *Y. pestis* individually as well as in two-plex assays at concentrations as low as 5 pg/mL and 10^6^ CFU/mL, respectively. We also introduce super avidin-biotin system (SABS) as a viable and effective means to enhance assay signal responses and lower detection limits. Together these technologies represent substantial advances in point-of-care and point-of-use detection applications.

## 1. Introduction

Microarrays have risen to the forefront of preferred analytical technologies for a variety of molecular applications including genetic studies, pathogen detection, and environmental sampling [1–6]. The key features of microarray technology are the exploitation of biological sensing elements for antigen recognition and the ability to screen those antigens against a vast array of probes. Although most arrays have traditionally relied on DNA-based sensing elements, advances in substrate and immobilization technology have made protein microarrays appealing for many sensing applications [7–11]. By combining the features of a protein-based antibody array into a single chip-based entity, CombiMatrix has created a powerful detection platform that lends itself well to point-of-use and point-of-care applications.

CombiMatrix semiconductor chips are comprised of 12,544 individually addressable microelectrodes that utilize complementary metal oxide semiconductor (CMOS) technology to measure the transduction of electrochemical signals resulting from an enzyme-enhanced electrochemical reaction following antigen binding to the appropriate recognition element. These chips are flexible in design and can be patterned with nucleic acid or protein recognition elements specific for a variety of antigens. CombiMatrix oligonucleotide arrays have been utilized in genotyping and gene expression assays [12], and antibody chips have been shown to successfully recognize a variety of analytes including saxitoxin and bacterial spores [13], the plant lectin ricin, M13 phage, and α−1 acid glycoprotein [14].

Although many detection platforms utilize fluorescence as a readout method, use of an enzymatic amplification step may improve detection due to the catalytic turnover of substrate. Amperometric electrochemicial detection as performed in the CombiMatrix ElectraSense® reader relies on the generation of electrons following the oxidation of substrate by horseradish peroxidase (HRP). This detection method has been shown to be both sensitive and specific in a variety of applications [12–15]. Amperometric-based sensors offer several advantages over other detection platforms including their cost-effectiveness, design flexibility, potential for miniaturization, and sensitivity [16].

Herein we describe the first use of the CombiMatrix antibody microarray designed towards the biological pathogens *Yersinia pestis*, *Bacillus anthracis*, and the bacterial toxin staphylococcal enterotoxin B (SEB). Using this platform, we were able to detect SEB and inactivated *Y. pestis* individually as well as in two-plex assays at concentrations as low as 5 pg/mL and 10^6^ colony-forming units (CFU)/mL, respectively. In addition, we introduce super avidin-biotin system (SABS) as a method to enhance electrochemical signal generation. SABS works by using high-affinity streptavidin (SA)-biotin binding to create layers of poly-HRP, resulting in the creation of an enzyme scaffold that amplifies signal intensity. This technique has widespread applicability and can be used in instances where it is necessary to boost the lower detection limit of an enzyme assay.

## 2. Results and Discussion

**Figure 1.**
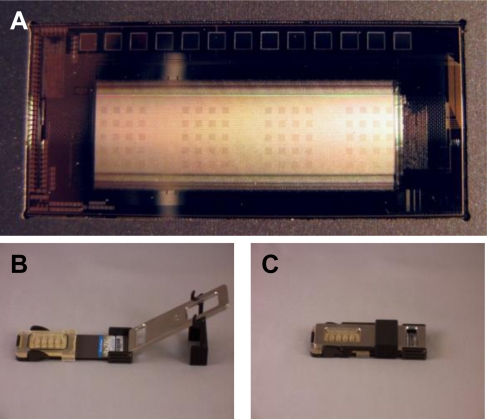
CombiMatrix antibody microarray (**A**) Arrays are subdivided into four identical sub-arrays divided into 20 discrete antibody blocks each; (**B**) Hybridization clamp that holds the chip and hybridization cap covering the microarray; (**C**) Fully assembled clamp, cap, and chip.

The CombiMatrix ElectraSense® microarray is mounted on a ceramic base the size of a microscope slide. Because the array has a platinum surface, it is resistant to most corrosive chemicals and supports the electrochemistries required for building customizable antibody or DNA microarrays as well as those for detecting macromolecular binding on the array. Following completion of the assay wet chemistry steps, microarrays are inserted into an ElectraSense^®^ reader for amperometric signal measurement at individual electrodes. For the purposes of our studies, we used CombiMatrix 4X2K chips that contained four identical sub-arrays of surface-immobilized antibodies designed to recognize the bacterial toxin SEB and pathogens, *B. anthracis* and *Y. pestis*. These capture antibodies function to bind target antigen on the chip surface. Following binding of antigen to the array, electrochemical detection is accomplished via the sequential addition of biotinylated detection antibodies, streptavidin-conjugated poly-horseradish peroxidase (PHRP-SA), and finally hydrogen peroxide and tetramethylbenzidine (TMB) substrate. PHRP-SA is a multimeric reagent that acts to amplify the detection signal by providing multiple HRP molecules for the oxidation of TMB substrate. The four sub-arrays contained antibodies for the three target antigens and were used in conjunction with a four-chamber hybridization cap that allowed for four independent assays on a single microarray. Each sub-array contained 20 antibody blocks, 5 for each immobilized antibody (15 total) and 5 for no-Ab negative controls. Each of the 5 blocks for a specific target contained 15 antibody-electrode pairs (75 total per sub-array). Using the CombiMatrix ElectroSense® reader, all four sub-arrays were measured simultaneously. An image of the CombiMatrix array and setup can be seen in Figure 1. For the purpose of our study, signals from an antigen-specific array must meet three criteria to be considered positive: (1.) a signal-to-noise (S/N) ratio of greater than 3; (2.) a signal-to-background (S/B) ratio of greater than 2; and (3.) a signal to “zero”(no antigen) (S/Z) ratio of greater than 2.

**Figure 2.**
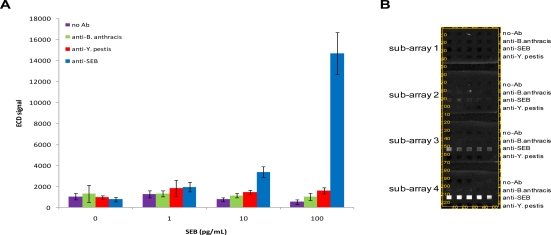
Electrochemical detection (ECD) assay for SEB. Antibody microarrays were incubated with 0 pg/mL, 1 pg/mL, 10 pg/mL, or 100 pg/mL SEB. Anti-*B. anthracis*, anti-*Y. pestis*, and anti-SEB refer to the antibodies immobilized on the chip surface. (**A**) Mean signals and SD; (**B**) Image of microarray.

We first characterized the detection limit of the chips for SEB by incubating arrays with 1–100 pg/mL SEB and interrogating with biotinylated anti-SEB. These experiments were designed to contain several controls for non-specific binding including a buffer control (no antigen present), surface negative control loci (no antibody present on surface), and non-specific antibody arrays. In these initial assays, SEB could be detected as low as 10 pg/mL (S/N = 15.03; S/B = 4.37; S/Z = 4.35) (Figure 2). Coefficients of variation (CVs) for SEB-specific loci generally ranged from 5 to 20%. Although CVs from microelectrodes directed against other targets (or no targets) were generally higher, the electrochemical signals from these latter loci were lower. Over the range of SEB concentrations tested, we did not observe the presence of non-specific positive signals using the criteria detailed above.

**Figure 3.**
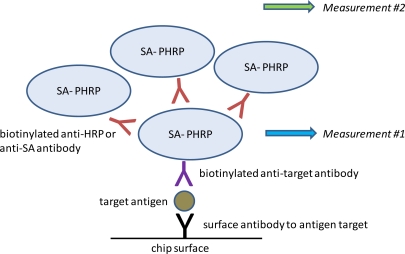
Schematic overview of SABS signal enhancement.

Having established the baseline limit of detection for SEB, we sought to improve assay sensitivity using a super avidin-biotin system (SABS). This system relies on creating layers of PHRP-SA scaffolding to increase signal generation (Figure 3). SABS can be accomplished using either biotinylated anti-SA or biotinylated anti-HRP antibodies to serve as poly-HRP-SA linkers. In direct comparisons, we found the former performed better than the latter in terms of specificity and amplitude of signal enhancement (data not shown) and was employed in subsequent experiments.

**Figure 4.**
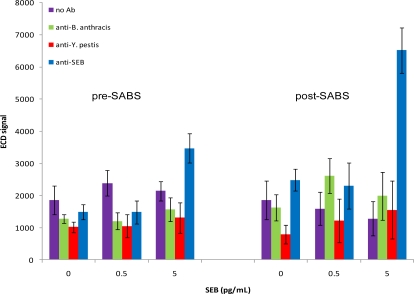
SABS assay for SEB. Chips were incubated with 0 pg/mL, 0.5 pg/mL, and 5 pg/mL. Anti-*B. anthracis*, anti-*Y. pestis*, and anti-SEB refer to the antibodies immobilized on the chip surface. Mean signals and SD are indicated.

To investigate the ability of SABS to enhance assay sensitivity for SEB, chips were incubated with 0, 0.5, 5, or 50 pg/mL SEB and experiments were performed as previously described up through the ElectraSense® measurement. We then added biotinylated anti-SA followed by PHRP-SA, TMB substrate, and measured again (Figure 4). If measured prior to incubation with biotinylated anti-SA (measurement #1), a noticeable signal from SEB was present at 5 pg/mL, although this increase was not deemed positive based on our pre-defined criteria (S/N = 4.39; S/B = 1.62; S/Z = 2.33). Signals from all other non-specific spots were not significantly different from background or noise levels based on the same criteria. Following SABS treatment (measurement #2), the detection limit for SEB improved to 5 pg/mL (S/N = 9.98; S/B = 5.07; S/Z = 2.63), suggesting that the SABS treatment was effective in enhancing signal generation, despite a general trend of higher standard deviations for SABS-treated samples. The increased CV values in these experiments were not surprising since any additional manipulations following the initial measurement have the potential for increasing overall variability at both target-specific and non-specific loci. Also, although we observed a small increase in signals non-specific loci following SABS treatment, these changes did not confound analysis, as they were much smaller than the SEB-specific signal amplification and did not generate any false positives based on the S/N, S/B, and S/Z criteria.

One aspect of the SABS experiments that warranted investigation was the possibility the first (pre-SABS) ECD measurement compromised the ability of SABS to enhance signal generation. We reasoned that if the TMB precipitates onto the chip surface during the first reading, it may foul the surface and prevent subsequent binding of biotinylated anti-SA to the PHRP-SA molecules. Comparison of a two-measurement protocol (as done in the previous experiment) with a single post-SABS measurement revealed that while the single measurement protocol produced higher signals overall, the patterns were not altered (data not shown).

**Figure 5.**
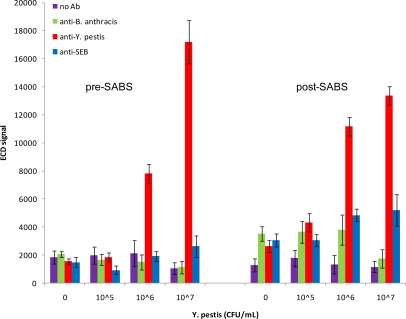
SABS assay for *Y. pestis*. Chips were incubated with 0, 10^5^, 10^6^, or 10^7^ CFU/mL inactivated *Y. pestis*, followed by interrogation with biotinylated anti-*Y. pestis*. Anti-*B. anthracis*, anti-*Y. pestis*, and anti-SEB refer to the antibodies immobilized on the chip surface. Mean signals and SD are indicated.

We next investigated the detection capabilities of the antibody microarray for inactivated *Y. pestis*. As can be seen in Figure 5, using a dilution series from 10^5^–10^7^ CFU/mL, we were able to detect *Y. pestis* as low as 10^6^ CFU/mL prior to SABS treatment (S/N = 6.06; S/B = 3.67; S/Z = 5.05). There was no improvement in the detection limit following SABS treatment; however, there was an improvement in most signal ratios (S/N = 14.81; S/B = 8.37; S/Z = 4.24). The increased background post-SABS inhibited detection of a positive signal at 10^5^ CFU/mL (S/N = 4.36; S/B=2.42; S/Z = 1.64). Although non-specific signal amplifications were observed for SEB and *B. anthracis*-specific loci following SABS treatment, these increases did not meet our criteria for positive signals. In contrast to the post-SABS results from SEB assays, the overall variability for target-specific and non-specific loci did not increase significantly after SABS amplification. Curiously, the signal from the most concentrated sample of *Y. pestis* (10^7^ CFU/mL) was lower than anticipated following SABS treatment. This has been periodically observed for SEB samples as well (data not shown) and may reflect a fouling of the chip surface by precipitated TMB that acts to inhibit diffusion of electroactive species to the array electrodes.

**Figure 6.**
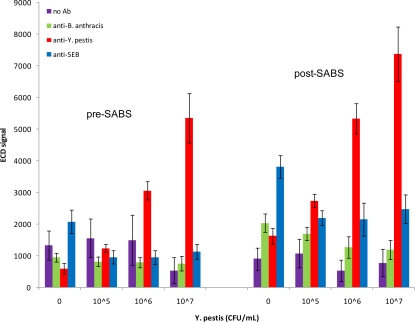
*Y. pestis* assay in 50% serum. Chips were incubated with 0, 10^5^, 10^6^, or 10^7^ CFU/mL inactivated *Y. pestis* diluted in 50% human serum. Anti-*B. anthracis*, anti-*Y. pestis*, and anti-SEB refer to the antibodies immobilized on the chip surface. Mean signals and SD are indicated.

Since all previous experiments were performed in buffer, we sought to emulate a more complex physiological environment by diluting antigens in the presence of 50% serum. In agreement with experiments performed in buffer, we observed a dose-response to spiked *Y. pestis* and positive detection at 10^6^ CFU/mL following SABS treatment (S/N = 14.49; S/B = 10.18; S/Z = 3.28) (Figure 6), although absolute signal intensity was noticeably blunted in the presence of 50% serum when compared to buffer. This was an improvement from the pre-SABS LOD of 10^7^ CFU/mL in 50% serum (S/N = 11.82; S/B = 10.11; S/Z = 9.05). Similar to previous SABS experiments, we observed some increase in signals from of non-specific loci, but none of the anti-*B. anthracis* or anti-SEB signals were positive based on our criteria; variability of these signals were not greatly affected by the presence of serum. In contrast, analogous experiments with SEB were found to be inconclusive, as signal levels were inconsistent and did not indicate any reproducible pattern (data not shown). Previous biosensor experiments for SEB detection in spiked serum samples have shown similar results [17]. The authors postulated that the presence of endogenous antibodies directed against SEB may have interfered with binding.

**Figure 7.**
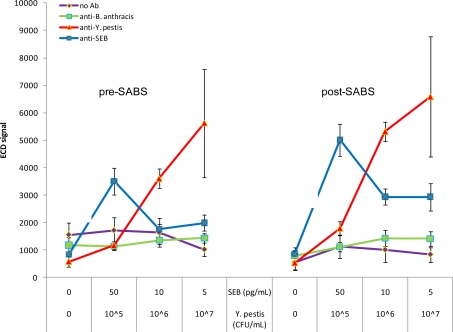
Two-plex assay for SEB and *Y. pestis*. Chips were incubated with 0, 10^5^, 10^6^, or 10^7^ CFU/mL *Y. pestis* and 0, 5, 10, or 50 pg/mL SEB. Anti-*B. anthracis*, anti-*Y. pestis*, and anti-SEB refer to the antibodies immobilized on the chip surface. Mean signals and SD are indicated. *Note*: breaks in SEB line serve as a reminder that concentrations for all samples are not continuous and that lines are only used to illustrate trends.

An important feature of detection systems is the ability to run multiplexed reactions for several targets simultaneously. CombiMatrix microarrays satisfy this requirement and are designed to recognize multiple targets tailored to the specific needs of the assay. For the purposes of this study, we chose to examine *Y. pestis* and SEB antigens in a multiplex format. First we ensured the absence of cross-reactivity or interference between anti-*Y. pestis* and anti-SEB antibodies. This was done by adding anti-*Y. pestis* antibodies to SEB assays and vice versa (data not shown). We next investigated the ability to perform two-plex assays by adding both *Y. pestis* and SEB to the same chips in reverse concentration patterns and interrogating with a mixture of anti-*Y. pestis* and anti-SEB antibodies. As can be seen in Figure 7, both *Y. pestis* and SEB can be detected on the same chip at 10^6^ CFU/mL (S/N = 3.80; S/B = 2.21; S/Z = 6.25) and 50 pg/mL (S/N = 3.74; S/B = 2.05; S/Z = 4.17), respectively. These results are in agreement with those previously observed for individual assays. Interestingly however, signal variability increased significantly at the highest *Y. pestis* concentration, but only on the *Y. pestis*-specific loci (∼10% CV to ∼35% CV); the cause for this effect is unknown. When exposed to SABS amplification, *Y. pestis* and SEB were detectable at 10^6^ CFU/mL (S/N = 9.54; S/B = 5.31; S/Z = 10.07) and 5 pg/mL (S/N = 7.77; S/B = 3.54; S/Z = 3.45), respectively. Again we observed a similar increase in *Y. pestis* signal variability at the highest concentration used. The lack of an observable decline in signal between 10 pg/mL and 5 pg/mL SEB is likely due to detection limitation rather than cross-reactivity, as supported by results in Figure 2 which shows there is not a large difference in signal between 1 pg/mL and 10 pg/mL SEB. Combined with the lack of “positive” results on the non-specific loci under these conditions, these results clearly demonstrate the multiplex capabilities of the CombiMatrix antibody microarray and the signal-enhancing properties of SABS.

The limits of detection (lowest positive concentration) for SEB attained with the CombiMatrix antibody microarray compare favorably with other commercial detection systems and laboratory-based assays. Enzyme-linked immunosorbent assays (ELISA) have been shown to detect as low as 0.5 ng/mL SEB [18]. Similarly, surface plasmon resonance (SPR) studies have recognized SEB as low as 0.5 ng/mL using a sandwich assay format and 5 ng/mL with direct detection [19]. While the Luminex flow cytometer (Luminex Corp., Austin, TX) is capable of detecting SEB at 50–100 pg/mL [20], it is still above the detection limits of the CombiMatrix system. In comparison to portable biosensors, the CombiMatrix system outperforms the fluorescence-based RAPTOR fiber optic biosensor (Research International, Seattle, WA) and the NRL Array Biosensor which are capable of detecting SEB at 0.5 ng/mL [21] and 0.1 ng/mL [22,23], respectively. In regards to *Y. pestis* detection, as performed, the CombiMatrix system was not as competitive as some other antibody-based assays. *Y. pestis* could be detected as low as 10^4^ CFU/mL using an up-converting phosphor technology (UPT)-based lateral-flow immunoassay [24], and a Luminex-based assay using as sandwich-assay format was able to detect aerosolized *Y. pestis* at 6 × 10^3^ CFU/mL [25].

Although the CombiMatrix antibody microarray system fares well overall in comparison with other detection systems, future assay optimization may enhance performance. One such potential improvement would be reducing assay time. Once the antibodies are patterned on the microarray, a typical assay takes about 3.5 h (5 h with SABS). Although most of these steps are not labor-intensive, if incubation steps could be shortened, the assay would become much more appealing to users desiring a rapid detection system. Another promising aspect for optimization is the employment of different detection antibodies. Replacement of the polyclonal antibodies used in these assays with monoclonal antibodies of higher sensitivity and selectivity may improve specific target detection and reduce background levels. In support of this possibility are previous findings that demonstrate the ability to enhance detection limits for SEB by using different SEB capture antibodies [22].

## 3. Experimental

### 3.1. Materials

CombiMatrix 4X2K antibody microarrays were supplied by CombiMatrix Corp [26]. These chips contained 4 identical sub-arrays, each of which contained 20 spots divided into 4 rows (*from top to bottom*: no antibody, anti-*B. anthracis*, anti-SEB, and anti-*Y. pestis* antibodies, with 5 replicate spots for each row). Rabbit polyclonal anti-SEB and anti-*Y. pestis* antibodies were obtained from the Department of Defense Critical Reagents Program (CRP) (Edgewood, MD). Biotinylated tracer antibodies were labeled using EZ link Sulfo-NHS-LC-biotin (Pierce, Rockford, IL). Biotinylated goat anti-streptavidin was purchased from Vector Laboratories Inc. (Burlingame, CA). Biotinylated rabbit anti-HRP was purchased from Jackson Immunoresearch (West Grove, PA). Poly-HRP40-streptavidin was obtained from Fitzgerald Industries International, Inc. (Concord, MA). SEB toxin was procured from Toxin Technologies (Sarasota, FL). Human sera were archived samples previously taken from healthy volunteers with informed consent. Inactivated *Y. pestis* was obtained from the Department of Defense Critical Reagents Program (CRP) (Edgewood, MD) and certified inactive prior to shipping. Technical grade casein was purchased from Sigma (St. Louis, MO). ElectraSense® TMB substrate and TMB rinse solutions were provided by CombiMatrix Corp. (Mukilteo, WA)

### 3.2. Methods

*ElectroSense® assay–*Arrays were hydrated in phosphate buffered saline/0.1% Tween-20 (PBST) for 20 min. Chips were then rinsed with phosphate buffered saline/0.3% casein/0.1% Tween-20 (PBSCT) and blocked with fresh PBSCT for 20 min. Following another rinse with PBSCT, SEB or inactivated *Y. pestis* antigen (diluted in PBSCT or 50% serum) was then applied for 1 hour. Chips were then rinsed 4 times with PBSCT then incubated with the appropriate antibody for 1 hour. Biotinylated SEB antibody was used at 2 μg/mL while anti-*Y. pestis* antibody was used at 4 μg/mL. Chips were rinsed 4 times with PBSCT then incubated with poly-HRP40-streptavidin (PHRP40-SA) for 30 min. Chips were then rinsed 5 times with PBSCT, once with PBS, then twice with TMB rinse. An amperometric electrochemical measurement was then taken following the addition of TMB substrate. All incubations were performed at room temperature on a rotating wheel unless otherwise indicated.

*Super avidin-biotin system (SABS)*–Following the first measurement as described above, chips were rinsed 4 times with PBSCT. Biotinylated goat anti-streptavidin was added at 10 μg/mL for 1 hour. Chips were then treated as above starting with 4 times PBSCT rinses and incubation with PHRP40-SA for 30 min.

*Statistical analysis*–For our purposes, three criteria were used to determine whether a signal was statistically significant (*i.e.*, positive): (1.) a signal-to-noise (S/N) ratio of greater than 3; (2.) a signal-to-background (S/B) ratio of greater than 2; and (3.) a signal to “zero”(*i.e.,* no antigen) (S/Z) ratio of greater than 2. The S/N ratio was defined as the [(mean antibody-specific array _x_ signal-mean buffer array _x_ signal) / SD of buffer array _x_ signal] and is commonly used to assess assay performance. The S/N ratio takes into account the standard deviation of the measurements; a S/N ratio of ≥3 was chosen to provide a 99% confidence interval. The S/B ratio was [mean antibody-specific array _x_ signal / mean buffer array _x_ signal]. This ratio controls for potential intra-sub-array variability, as both the no-Ab and antibody-specific signals were from the same sub-array. The S/Z ratio is described as [mean antibody-specific array _x_ signal / mean antibody-specific array _buffer_ signal], where the denominator is the mean signal from the buffer condition array for that specific antibody. The S/Z ratio is important to compare different sub-arrays on the same chip because the buffer (no antigen) condition was performed on a different sub-array then the antigen dose conditions. We empirically determined that the combination of these threshold conditions yielded the highest level of sensitivity while minimizing the potential for false positives (data not shown), thus all three criteria must be met for a signal to be considered positive.

## 4. Conclusions

Herein we describe application of the CombiMatrix antibody microarray system designed for detection of the bacterial pathogens *B. anthracis, Y. pestis*, and SEB. Using a protocol involving the use of polymeric HRP-SA conjugate, we were able to detect SEB and *Y. pestis* as low as 5 pg/mL and 10^6^ CFU/mL, respectively, in assays incorporating a SABS amplification step. We demonstrate the ability to detect these pathogens individually as well as in a two-plex assay format. In addition to its ease-of-use and portability, the versatility, flexibility, and sensitivity of the CombiMatrix technology make it an attractive solution to the detection needs of a variety of sensor-based areas including disease diagnosis, genetic screenings, and biological/chemical warfare (BCW) threat applications.
